# Lipidomics of familial longevity

**DOI:** 10.1111/acel.12064

**Published:** 2013-04-02

**Authors:** Vanessa Gonzalez-Covarrubias, Marian Beekman, Hae-Won Uh, Adrie Dane, Jorne Troost, Iryna Paliukhovich, Frans M Kloet, Jeanine Houwing-Duistermaat, Rob J Vreeken, Thomas Hankemeier, Eline P Slagboom

**Affiliations:** 1 Netherlands Metabolomics CentreLeiden, The Netherlands; 2 Analytical Biosciences, Leiden UniversityLeiden, The Netherlands; 3 Molecular Epidemiology, Leiden University Medical CenterLeiden, The Netherlands; 4 Netherlands Consortium for Healthy AgeingLeiden, The Netherlands; 5 Medical Statistics and Bioinformatics, Leiden University Medical CenterLeiden, The Netherlands

**Keywords:** aging, gender differences, human, longevity, mass spectrometry, oxidative stress

## Abstract

Middle-aged offspring of nonagenarians, as compared to their spouses (controls), show a favorable lipid metabolism marked by larger LDL particle size in men and lower total triglyceride levels in women. To investigate which specific lipids associate with familial longevity, we explore the plasma lipidome by measuring 128 lipid species using liquid chromatography coupled to mass spectrometry in 1526 offspring of nonagenarians (59 years ± 6.6) and 675 (59 years ± 7.4) controls from the Leiden Longevity Study. In men, no significant differences were observed between offspring and controls. In women, however, 19 lipid species associated with familial longevity. Female offspring showed higher levels of ether phosphocholine (PC) and sphingomyelin (SM) species (3.5–8.7%) and lower levels of phosphoethanolamine PE (38:6) and long-chain triglycerides (TG) (9.4–12.4%). The association with familial longevity of two ether PC and four SM species was independent of total triglyceride levels. In addition, the longevity-associated lipid profile was characterized by a higher ratio of monounsaturated (MUFA) over polyunsaturated (PUFA) lipid species, suggesting that female offspring have a plasma lipidome less prone to oxidative stress. Ether PC and SM species were identified as novel longevity markers in females, independent of total triglycerides levels. Several longevity-associated lipids correlated with a lower risk of hypertension and diabetes in the Leiden Longevity Study cohort. This sex-specific lipid signature marks familial longevity and may suggest a plasma lipidome with a better antioxidant capacity, lower lipid peroxidation and inflammatory precursors, and an efficient beta-oxidation function.

## Introduction

The human plasma lipidome is composed of thousands of different lipids, whose diversity in function is only paralleled by its wide variation in structure (Quehenberger *et al*., 2010). The current increase in patients with lipid-related disorders has intensified the focus in lipid metabolism, dissecting it into its major classes and its connection with signaling pathways in age-related diseases (Wang *et al*., 2011; Sugiyama & Agellon, 2012). The molecular composition and concentration of lipid species determine their cellular location, residence time, metabolism, and consequently their impact in disease and health. For example, LDL particles are the main carriers of sphingomyelins and ceramides, while ether phosphocholines are mainly present in HDL particles, partly explaining their opposing roles in atherogenesis (Nelson *et al*., 2006; Yeboah *et al*., 2010). Molecular characterization of triglyceride and sphingomyelin species has identified specific lipids underlying insulin resistance (Suhre *et al*., 2010; Rhee *et al*., 2011), Alzheimer's (Han *et al*., 2011), and obesity (Pietilainen *et al*., 2007). Moreover, depletion of ether phosphocholine species in plasma has been associated with hypertension (Graessler *et al*., 2009) and diabetes (Sysi-Aho *et al*., 2011). Hence, the field of metabolomics may aid to better understand health and disease by pinpointing specific metabolites and pathways involved. Up-to-date, lipidomics has been used as a powerful tool from the perspective of disease risk and treatment efficacy (Laaksonen *et al*., 2006), but not for the identification of lipid metabolites associated with longevity and healthy aging. Classical lipid parameters such as total triglyceride levels, HDL, and LDL particle size have shown to associate with human familial longevity and healthy aging (Barzilai *et al*., 2003; Heijmans *et al*., 2006). Moreover, a higher ratio of monounsaturated (MUFA) over polyunsaturated (PUFA) lipids has been suggested as a marker of longevity because a small study indicated that erythrocyte membranes of offspring of nonagenarians have a higher content of MUFA and lower content of PUFA species (Caprari *et al*., 1999; Puca *et al*., 2007). Therefore, lipidomics may contribute to gain new insights into the mechanisms underlying human longevity and healthy aging.

Using the unique design of the Leiden Longevity Study, we have previously identified that offspring of nonagenarians show a lower prevalence of insulin resistance, hypertension, and cardiovascular disease (Barzilai *et al*., 2001; Atzmon *et al*., 2004; Schoenmaker *et al*., 2006; Westendorp *et al*., 2009) accompanied by lower levels of total triglycerides and larger LDL particles, which are indicative markers of healthy aging at middle age (Heijmans *et al*., 2006; Vaarhorst *et al*., 2011). Here, we used a lipidomic-targeted approach to extend these investigations and found a novel profile of lipid species associated with familial longevity independent of previously identified parameters such as total triglyceride concentrations. We assessed the influence of age and sex on the longevity-associated lipids, observing a youthful profile for female offspring. As our study has a considerable sample size, we also evaluated whether the ratio of monounsaturated over polyunsaturated of the 19 longevity lipids was associated with familial longevity. Moreover, the longevity-associated lipid profile was also correlated with classical lipid parameters including total cholesterol, HDL-C, LDL-C, lipoprotein particle size, and the prevalence of hypertension, and diabetes type 2. Our observations confirmed and extended the notion that lipid metabolism is involved in familial longevity in a sex-dependent manner.

## Results

We successfully obtained lipidomic profiles consisting of 128 different lipid species in 1526 offspring and 675 controls. Population characteristics in Table 1 show that offspring and controls were similar in terms of age, BMI, and most classical lipid parameters except for total triglycerides and the TG-HDL-C ratio whose values were higher in controls (*P* < 0.005). Also, compared to the offspring of nonagenarians, controls were more likely to present diabetes and hypertension. Demographic differences between men and women are presented in Table S1.

**Table 1:** Demographics of the study population

	Controls (675)	Offspring (1526)
*N*	Males (285), Females (390)	Males (714), Females (812)
Age (years)	58.8 ± 7.4 (30.2–79.2)	59.4 ± 6.6 (33.6–80.3)
BMI (kg m^−2^)	25.3 ± 3.6 (25.3–25.9)	25.8 ± 3.6 (25.2–25.5)
Total TG (mm)	1.94 ± 1.37 (1.81–2.03)	1.76 ± 1.05 (1.69–1.80)
HDL-C (mm)	1.43 ± 0.465 (1.40–1.47)	1.45 ± 0.448 (1.43–1.48)
LDL-C (mm)	3.36 ± 0.938 (3.29–3.43)	3.34 ± 0.987 (3.29–3.39)
HDL particle size (nm)	9.01 ± 0.509 (8.99–9.10)	9.05 ± 0.507 (9.03–9.08)
LDL particle size (nm)	21.1 ± 0.838 (21.09–21.21)	21.3 ± 0.826 (21.2–21.3)
TG/HDLC ratio	1.66 ± 1.54 (1.54–1.78)	1.45 ± 1.27 (1.39–1.52)
Diabetes type 2 (%)*	47 (8.2)	56 (4.3)
Hypertension (%)†	166 (28.7)	288 (22.3)
MUFA-to-PUFA ratio†	0.881 ± 0.37 (0.851–0.911)	0.928 ± 0.380 (0.907–0.948)

Data are reported as mean values ± SD followed by range values in parentheses.

* Values are number of cases followed by percentage in parenthesis.

† Values are adjusted means from the regression model ± SD followed by range values between parenthesis.

### Lipid species associate with familial longevity in females only

**Fig. 1:**
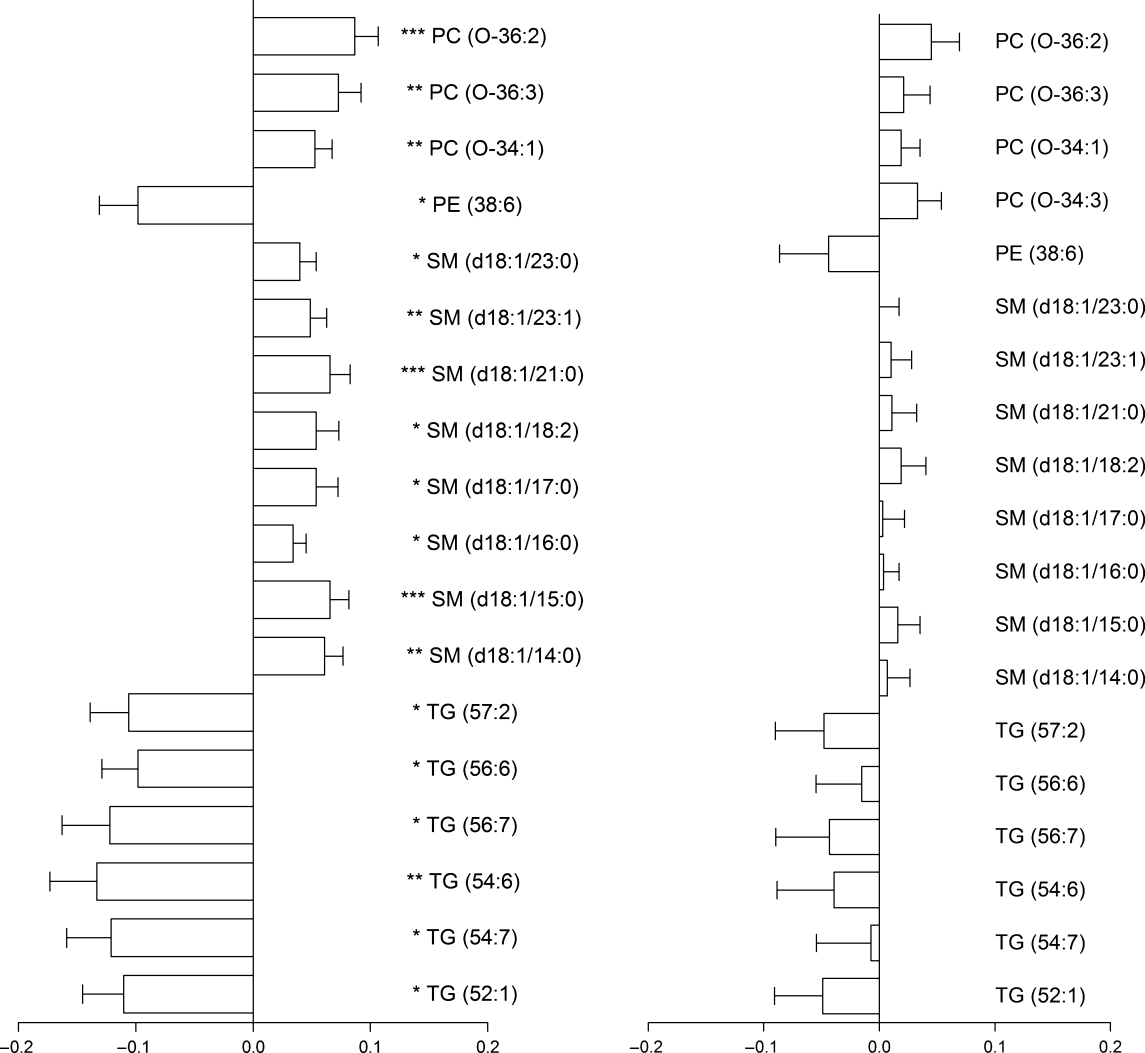
Differences in lipid species between offspring of nonagenarians and controls. Left, differences in women; Right, differences in men. Statistical significance was observed in females only (*P < 0.005; **P < 0.001; ***P < 0.0005). *X*-axis depicts the effect size of levels of ln-transformed lipid species ± robust standard errors. Positive values indicate lipid levels higher in offspring. Negative values indicate lipid levels lower in offspring compared with controls.

Relative lipid levels were calculated for a total of 128 lipids from six different classes: TG (*n* = 52), SM (*n* = 17), PE (*n* = 7), cholesteryl esters (*n* = 4), lysophosphatidylcholines (*n* = 16), and PC (*n* = 32). We compared the relative levels of these 128 lipids between offspring of nonagenarians and controls to identify lipid species that associate with familial longevity. In men, lipidomic differences between offspring and controls did not reach statistical significance. However, in women, we identified 19 lipids that significantly associated with familial longevity (Fig. 1). Relative levels of ether PC and SM species were higher (3.5–8.7%), while long-chain triglycerides and PE (38:6) were lower (9.4–12.4%) in the offspring of nonagenarians compared with controls (Table S2). TG (54:6) and PC (O-36:2) species showed the largest differences between offspring and controls with 12.4% lower and with 8.7% higher levels in female offspring. In men, we obtained similar results for ether PC and TG species, but effect sizes between offspring and controls were smaller than in women and differences did not reach statistical significance (Table S3).

Total triglycerides have been previously reported as a determinant of familial longevity in women of the Leiden Longevity Study (Vaarhorst *et al*., 2011). Regression analyses accounting for total triglycerides showed that the association with familial longevity remained significant for six lipid species (*P* < 0.005). Female longevity in LLS families was associated with ether PC (O-36:2), PC (O-36:3), SM (d18:1/14:0), SM (d18:1/15:0), SM (d18:1/21:0), and SM (d18:1/23:1) independent of total TG (*P* < 0.005, Table S4). In addition, we investigated whether lipid levels in female offspring and controls were affected by age. Nine of the 19 lipid species either increased or decreased with age, but most of them were not affected by age after 55 years, except for SM (d18:1/23:1) and TG (54:6), which showed differential levels before and after 55 years in female offspring but not in controls (Tables 2 and 3).

**Table 2:** Effect of age (men and women) and sex in lipid species that associated with familial longevity

Lipid	Adjusted mean†	Effect of age		Effect of sex	
	
		Beta ± SE‡	*P*-value*	Beta ± SE§	*P*-value*
PC (O-34:3)	0.736	−0.067 ± 0.010	1.0 × 10^−11^	−0.134 ± 0.012	<10^−20^
PC (O-34:1)	0.41	−0.021 ± 0.008	7.0 × 10^−3^	−0.121 ± 0.010	<10^−20^
PC (O-36:3)	0.176	−0.041 ± 0.011	1.3 × 10^−4^	−0.096 ± 0.013	1.3 × 10^−12^
PC (O-36:2)	0.122	−0.029 ± 0.011	1.1 × 10^−2^	−0.098 ± 0.014	6.5 × 10^−12^
PE (38:6)	0.819	0.046 ± 0.019	1.3 × 10^−2^	−0.221 ± 0.026	4.3 × 10^−17^
SM (d18:1/14:0)	0.362	0.039 ± 0.009	2.2 × 10^−5^	−0.154 ± 0.011	<10^−20^
SM (d18:1/15:0)	0.229	0.050 ± 0.009	5.0 × 10^−8^	−0.177 ± 0.011	<10^−20^
SM (d18:1/16:0)	6.624	0.012 ± 0.006	3.9 × 10^−2^	−0.969 ± 0.008	<10^−20^
SM (d18:1/17:0)	0.085	0.056 ± 0.010	1.0 × 10^−8^	−0.190 ± 0.012	<10^−20^
SM (d18:1/18:2)	0.023	0.003 ± 0.010	7.8 × 10^−1^	−0.274 ± 0.013	<10^−20^
SM (d18:1/21:0)	0.282	0.021 ± 0.011	3.3 × 10^−2^	−0.210 ± 0.013	<10^−20^
SM (d18:1/23:1)	0.501	0.033 ± 0.008	8.1 × 10^−5^	−0.251 ± 0.010	<10^−20^
SM (d18:1/23:0)	0.74	0.002 ± 0.008	7.5 × 10^−1^	−0.144 ± 0.010	<10^−20^
TG (52:1)	4.065	0.073 ± 0.018	7.2 × 10^−5^	0.225 ± 0.026	2.4 × 10^−17^
TG (54:7)	0.376	0.063 ± 0.021	3.0 × 10^−3^	0.144 ± 0.028	4.7 × 10^−7^
TG (54:6)	1.38	0.043 ± 0.023	5.7 × 10^−2^	0.222 ± 0.031	8.6 × 10^−13^
TG (56:7)	1.021	0.052 ± 0.020	1.0 × 10^−2^	0.192 ± 0.028	1.8 × 10^−11^
TG (56:6)	1.116	0.041 ± 0.018	2.3 × 10^−2^	0.157 ± 0.024	4.9 × 10^−11^
TG (57:2)	1.216	0.059 ± 0.018	1.0 × 10^−3^	0.219 ± 0.025	1.7 × 10^−18^

* Nominal *P*-value.

† Back-transformed adjusted means of relative lipid levels.

‡ Beta, effect size of age in 10 years and robust standard error.

§ Beta, effect size of sex and robust standard error; negative values indicate lipid levels lower in men, positive values indicate lipid levels higher in men.

**Table 3:** Effect of age after 55 years on levels of lipid species that associated with familial longevity

	Controls		Offspring	
		
	Beta ± SE †	*P*-value*	Beta ± SE†	*P*-value*
PC (O-34:3)	0.037 ± 0.322	0.909	0.034 ± 0.369	0.927
PC (O-34:1)	−0.384 ± 0.303	0.206	−0.124 ± 0.236	0.600
PC (O-36:3)	0.015 ± 0.372	0.969	−0.352 ± 0.372	0.345
PC (O-36:2)	0.029 ± 0.386	0.940	−0.718 ± 0.428	0.094
PE (38:6)	−0.900 ± 0.680	0.186	0.602 ± 0.652	0.356
SM (d18:1/14:0)	−0.051 ± 0.278	0.854	0.524 ± 0.288	0.069
SM (d18:1/15:0)	−0.368 ± 0.296	0.215	0.408 ± 0.274	0.136
SM (d18:1/16:0)	−0.049 ± 0.221	0.825	0.344 ± 0.185	0.064
SM (d18:1/17:0)	−0.118 ± 0.329	0.719	−0.002 ± 0.448	0.996
SM (d18:1/18:2)	−0.589 ± 0.404	0.146	0.093 ± 0.341	0.786
SM (d18:1/21:0)	−0.058 ± 0.319	0.855	0.418 ± 0.315	0.186
SM (d18:1/23:1)	−0.169 ± 0.283	0.550	0.682 ± 0.226	0.003
SM (d18:1/23:0)	0.197 ± 0.255	0.440	0.630 ± 0.238	0.008
TG (52:1)	−0.050 ± 0.691	0.942	1.40 ± 0.632	0.027
TG (54:7)	−0.672 ± 0.786	0.393	1.24 ± 0.637	0.052
TG (54:6)	−0.360 ± 0.793	0.650	1.86 ± 0.690	0.005
TG (56:7)	−0.686 ± 0.727	0.346	1.10 ± 0.656	0.094
TG (56:6)	−0.699 ± 0.609	0.252	1.18 ± 0.537	0.028
TG (57:2)	−0.779 ± 0.633	0.219	0.828 ± 0.544	0.129

* Nominal *P*-value.

† Beta, effect size of age after 55 years and robust standard error; negative values indicate lipid levels decrease and positive values indicate lipid levels increase after 55 years.

### Female offspring of nonagenarians showed a higher MUFA-to-PUFA ratio

**Fig. 2:**
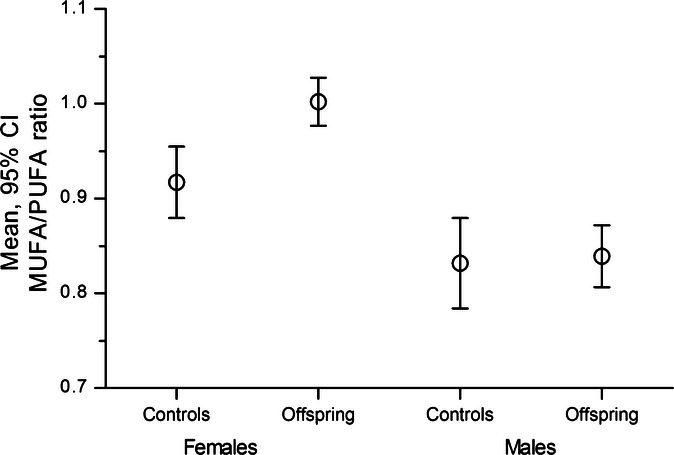
MUFA-to-PUFA ratio differences in offspring of nonagenarians and controls. Adjusted mean values of MUFA-to-PUFA ratios and 95% confidence intervals; female differences *P* = 2.6 × 10^−4^; male differences *P* > 0.05.

Differences in content of polyunsaturated (PUFA) and monounsaturated (MUFA) lipids determine membrane peroxidation, and the MUFA-to-PUFA ratio has been suggested as a marker of longevity (Pamplona *et al*. 1998; Caprari *et al*., 1999). Therefore, we determined differences in the MUFA-to-PUFA ratio between offspring and controls for each one of the 19 lipid species that associated with familial longevity. Offspring of nonagenarians displayed a higher MUFA-to-PUFA ratio compared with controls (Fig. 2), and these differences were statistically significant in women only. Compared to controls, female offspring showed a 9.2% higher MUFA-to-PUFA ratio (MUFA-to-PUFA ratio controls: 0.917, CI: 0.880–0.955 versus MUFA-to-PUFA ratio offspring: 1.001, CI: 0.977–1.027; *P*-value = 2.6 × 10^−4^).

### Effect of sex and age on lipids associated with familial longevity

We observed several lipidomic differences between sexes. All long-chain TG species were lower in women compared to men particularly TG (52:1), TG (54:6), and TG (57:2), while PE (38:6), ether PC, and SM species were higher in women (*P* < 0.005). The largest differences between sexes were observed for all SM, PC (O-34:1), and PC (O-34:3) species, which were higher in women (Table 2).

Next, we explored the effect of chronological age on these 19 lipid species. We detected a significant association between age and levels of nine of the 19 lipids. Most ether PC species showed a negative association, while all other longevity-associated lipids displayed a positive association with age, except for SM (d18:1/18:2) and (d18:1/23:0), which were not significantly affected by age. The most significant effect of age was observed for ether PC (O-34:3) and (O-36:3) showing negative and TG (52:1), (54:7), and (57:2) showing positive associations with age (Tables 2 and S5). In addition, Pearson's correlation coefficients indicated a linear correlation with age for 10 and 13 lipid species in female controls and offspring, respectively (*R* < 0.25, *P* < 0.005; Table S6). When considering age after 55 years, only two lipid species, SM (d18:1/23:1) and TG (54:6), were affected by age (Table 3). A summary of the linear parameters of the effect of age after 55 years on these 19 lipid species is listed in Table S6. Our results indicate that higher levels of ether PC and lower levels of PE (38:6) and TG species are representative of a youthful profile, supporting the notion that offspring of nonagenarians have a healthy attenuated aging process (Westendorp *et al*., 2009).

### Correlation between longevity-associated lipids, classical lipids, and clinical parameters

**Fig. 3:**
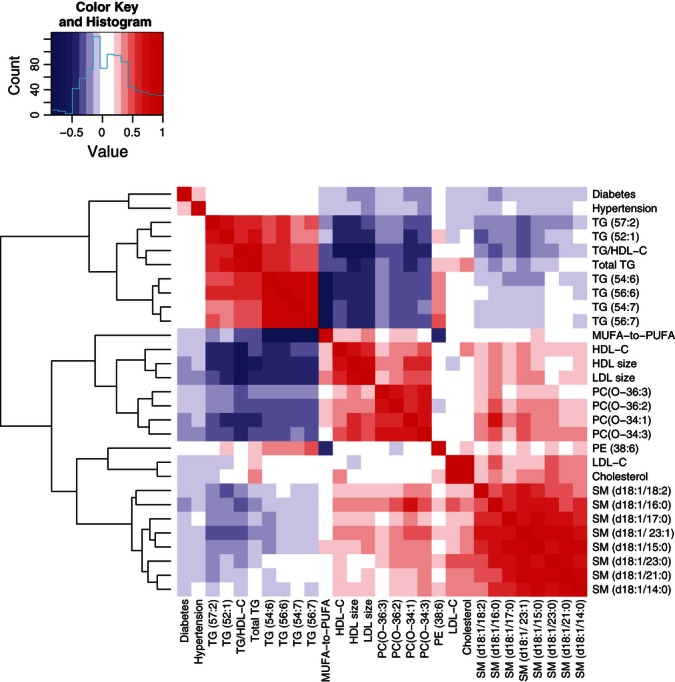
Correlation heatmap of longevity-associated lipids and clinical parameters in female offspring.

We investigated potential correlations between the 19 longevity-associated lipids and classical lipid parameters including total triglycerides, HDL-C, LDL-C, lipoprotein particle size, the TG-HDL-C and MUFA-to-PUFA ratios, and the prevalence of hypertension or diabetes. Besides the expected correlations among lipids of similar classes shown as highly correlated clusters in Fig. 3, we also detected lipidomic correlations among lipid species not previously reported. First, PC (O-34:1) was positively correlated with SM (d18:1/16:0) and negatively to TG (57:2), (54:6), and (52:1). Similarly, SM (d18:1/23:1) and (d18:1/18:2) negatively correlated with TG (57:2) and (52:1).

Among classical lipid parameters, total triglycerides negatively correlated with HDL-C, lipoprotein particle size, SM (d18:1/23:1), SM (d18:1/16:0), and most ether PC, particularly PC (O-34:3) and PC (O-34:1). In addition, HDL particle size was positively correlated with PC (O-34:3) and PC (O-34:1), while LDL and HDL particle size inversely correlated with TG (54:6) and TG (56:6), and with the TG-HDL-C ratio.

The MUFA-to-PUFA ratio showed an inverse correlation to PE (38:6) and several TG species and was positively correlated with lipoprotein particle size, HDL-C, SM (d18:1/15:0), ether PC (O-36:2), and PC (O-34:1).

As recent investigations have pinpointed specific lipid species linked to hypertension and diabetes, we sought to identify correlations between the 19 lipid species and the prevalence of these diseases. Ether PC (O-34:1), PC (O-34:3), SM (d18:1/16:0), and SM (d18:1/23:1) showed a prominent inverse correlation with diabetes type 2 and in a similar manner PC (O-34:1), PC (O-34:3), and PC (O-36:1) with hypertension.

## Discussion

The role of lipids in metabolic health and longevity has become a matter of intense research because several lipid parameters are used as surrogate markers for age-related diseases including hypertension, diabetes type 2, and cardiovascular disease (Yeboah *et al*., 2010; Boullart *et al*., 2012; Miller *et al*., 2011). Therefore, we investigated the relationship between 128 lipid species from the plasma lipidome and familial longevity in offspring of nonagenarians. Sex differences in lipid profiles have been well established (Wang *et al*., 2011), hence we investigated the association of the plasma lipidome with familial longevity separately in men and women. In men, PC, PE (38:6), and TG species differed between offspring and controls, but differences did not reach statistical significance. In women, we identified a profile of 19 lipid species of the SM, PC, PE, and TG classes associated with familial longevity. This lipid profile was characterized by a higher MUFA-to-PUFA ratio. After adjusting for total triglyceride levels, a major lipid determinant of familial longevity in women (Vaarhorst *et al*., 2011), two ether PC (O-36:3), PC (O-36:3), and four SM species, SM (d18:1/14:0), SM (d18:1/15:0), SM (d18:1/21:0), and SM (d18:1/23:1) maintained its association with familial longevity. This is the first study to report in a large population plasma lipidomic profiles and its association with familial longevity in middle age. The lipid profile, representative of healthy aging, consisted of higher levels of ether PC and SM species and lower levels of PE (38:6) and long-chain TG species.

Sphingomyelin species are structural components of tissue membranes, important cellular messengers, and are abundant in nerve cells. Low levels of SM species have been associated with neurodegenerative diseases including Alzheimer's, Parkinson's, Huntington's (Piccinini *et al*., 2010), and with metabolic disorders such as diabetes (Suhre *et al*., 2010), subclinical atherosclerosis, (Nelson *et al*., 2006), and cardiovascular disease (Holland & Summers, 2008). The fate of SM species can be either hydrolysis into ceramides by sphingomyelinase (SMase), or deacylation into sphingosine phosphorylcholine (SPC). Little is known about the latter pathway, SPC is believed to be a signaling molecule present in plasma in low concentrations with a very short half-life (Nixon *et al*., 2008). Conversion of SM into ceramides may occur in plasma; secretory SMAase (sSMase) hydrolyzes lipoprotein-bound SM at neutral pH preceding a signaling cascade, aggregating LDL or reorganizing lipid rafts in the plasma membrane (Milhas *et al*., 2010). SMase activity tends to increase with age decreasing SM levels and increasing ceramide levels (Petkova *et al*., 1988; Smith *et al*., 2006; Smith & Schuchman, 2008). This lead us to hypothesize that higher levels of SM species in female offspring may be a consequence of lower sSMase activity possibly decreasing the risk of ceramide-related diseases. However, a role of SM deacylation in the maintenance of higher levels of SM in offspring could not be discarded. Future studies measuring a coordinated decrease in ceramide with increased SM levels are warranted.

We observed that lipid species that may represent a healthy profile tend to decrease with age (ether PC), while lipids associated with aging diseases tend to increase with age (TG), but levels of potentially beneficial SM species also increased with age in controls and female offspring showed the highest levels (Tables 2 and S5). Hence, higher levels of SM species may not necessarily be representative of a youthful profile. Alternatively, higher levels of SM species may be compensating for the deleterious increase in TG with age in females. In support to this hypothesis, we observed that in women older than 55 years, SM species increased with age in controls (higher slope), but did not show a significant change with age in offspring, and in contrast, levels of TG species showed a slower increase in female offspring compared with controls (lower slope, Table S6). On average, however, levels of SM species were higher in offspring women regardless of age.

Ether PC species have been found to prevent oxidation of polyunsaturated fatty acids in lipoproteins, a feature suggested as sex dependent (Helmy *et al*., 2003; Wiesner *et al*., 2009). Ether PC species were present in higher levels in the offspring of nonagenarians a characteristic of a youthful profile, which is consistent with their antioxidant and cardioprotective roles. In addition, down-regulation of ether phospholipids is linked to the prognosis of hypertension and diabetes (Graessler *et al*., 2009; Suhre *et al*., 2010). Thus, it is possible that higher levels of ether phosphocholines in female offspring reflect a better antioxidant capacity and a healthier lipid profile compared to that of controls.

PE species, the second most abundant membrane phospholipid in mammals, have been identified as modulators of inflammation and apoptosis (Gibellini & Smith, 2010), but little is known about specific molecular species. PE (38:6), which was present in lower levels in female offspring, is a highly polyunsaturated phospholipid that can bear different acyl chains including several proinflammatory precursors. Lower levels of this particular PE may be protecting offspring females against peroxidation of fatty acids and generation of proinflammatory molecules such as arachidonic acid (C20:4).

High TG levels have been associated with the presence of TG-rich lipoproteins and their remnants, which participate in the development of atherogenesis and cardiovascular disease. Moreover, total TG concentrations increase significantly with age in women after menopause (Sugiyama & Agellon, 2012). Nevertheless, the molecular characterization of individual triglyceride molecules involved in atherogenesis and increasing age in women remains elusive (Schwartz & Reaven, 2012). Four of the triglyceride species of the longevity profile, TG (54:7), TG (54:6), TG (56:7), and TG (56:6), are long-chained and highly polyunsaturated, and thus, they are considered highly peroxidizable. Typically, long-chain triglycerides undergo beta-oxidation in mitochondria or peroxisomes whose enzymatic capacity decreases with age. Deleterious PE (38:6) and most TG species increased at a slower rate in female offspring after 55 years compared with controls. Therefore, lower levels of unsaturated TG species in female offspring may reflect an efficient beta-oxidation function compared to controls, a tendency that may be opposing the effects of age.

The fact that the longevity lipid profile was characterized by a higher MUFA-to-PUFA ratio is consistent with reports showing a negative correlation between membrane double bond content and longevity in mammals (Pamplona *et al*., 2000; Mitchell *et al*., 2007). As lower PUFA levels may lead to decreased lipid peroxidation and lower oxidative damage (Pamplona *et al*., 2002; Puca *et al*., 2007), the plasma lipidome of female offspring in contrast to that of controls may be less prone to lipid peroxidation. These observations may be indicative of a shift from polyunsaturated to monounsaturated lipid species or differential unsaturated lipid metabolism in long-lived families. Together with a higher presence of scavenger ether PC and lower polyunsaturated TG species, the plasma lipidome of female offspring may reflect an efficient metabolism of different lipid species as a synchronized attempt to maintain a low oxidative environment.

Classical lipid differences between men and women have long been recognized, but lipid differences between sexes at the molecular level are rather limited (Sugiyama & Agellon, 2012). For example, it is well known that total TG concentrations are higher in men than in women. However, the plasma lipidome contains dozens of different triglyceride species that constitute the parameter of total TG (Quehenberger *et al*., 2010; Quehenberger & Dennis, 2011). Gender lipidomic differences also recognize an increase in total TG concentrations in women after menopause accompanied by higher transcription and expression of lipid trafficking and oxidation genes, and these enzymatic changes with age have not been observed in men (Miller *et al*., 2011; Sugiyama & Agellon, 2012). This study provides in-depth gender differences including higher levels of TG (52:1) and TG (54:6) in men compared to women. Although age >55 years affected only two lipid species, the linear relationship of age and lipid levels showed differences between offspring and controls in females (Table S6), suggesting that it is possible that age and maybe menopause, a sex-specific event, may enhance differences between offspring and controls in women partly explaining the lack of lipidomic differences in men. Other possible explanations for the observed sex differences include dissimilarities in body fat between men (10–15%) and women (15–20%) and differential hormonal regulation of lipid metabolism. For example, estrogen, which decreases with age, has a protective effect on women's cardiovascular health. The consequences and mechanisms underlying this protective effect are not clear. However, a recent study showed that a decrease in estrogen enhances the accumulation of visceral fat in women through aldh1a1, but the effect of the enzyme was not relevant in men, supporting sex differences in lipid metabolism driven by hormones (Yasmeen *et al*., 2013). Accordingly, if delayed menopause is a characteristic of female longevity (Ossewaarde *et al*., 2005), then it is possible that fewer offspring women in the LLS have undergone menopause showing a healthier plasma lipid profile.

Finally, we investigated which of these 19 longevity-associated lipids correlated with hypertension or diabetes, because a low prevalence of these diseases are markers of familial longevity (Rozing *et al*., 2010). We observed that PC (O-34:1), PC (O-34:3), SM (d18:1/16:0), and SM (d18:1/23:1) negatively correlated with type 2 diabetes and likewise PC (O-36:2), PC (O-34:1), and PC (O-34:3) with hypertension. These results are in agreement with previous reports showing a decrease in ether PC species in hypertensive patients parallel to an increase in long-chain polyunsaturated PE and TG species (Graessler *et al*., 2009). Similarly, our findings confirm former investigations showing that several SM species, including those identified here, are deregulated in diabetes (Suhre *et al*., 2010; Sysi-Aho *et al*., 2011). Together, these observations provide an overlapping lipid profile between diabetes and hypertension encompassing most ether PC and SM species associated with familial longevity. Investigation of the cellular roles and tissue localization of these ether PC and SM species would aid to understand the molecular mechanisms by which these lipids contribute to familial longevity and healthy aging. Ongoing endeavors are also being directed to further understand differences between offspring and controls and to dissect sex differences in the plasma lipidome. Future studies would confirm our observations in other populations and its association with health parameters.

In conclusion, we have identified 19 lipid species associated with female familial longevity in middle age including higher levels of ether PC and SM and lower levels of PE (38:6) and long-chain TG species, a profile characterizing the offspring of nonagenarians, a group prone to healthy aging. Ether PC and SM species were identified as novel longevity markers in women, independent of total triglycerides levels. Several longevity-associated lipids correlated with a lower risk of hypertension and diabetes in the Leiden Longevity Study cohort. This lipid profile marks familial longevity and may suggest a plasma lipidome with a better antioxidant capacity, lower lipid peroxidation and inflammatory precursors, and an efficient beta-oxidation function.

## Experimental procedures

### Participants

In 2001, The Leiden Longevity Study (LLS) recruited nonagenarian siblings of Caucasian descent (956 families; age, 90–104 years), 1671 of their offspring and 744 partners of the offspring as controls. The study protocol was approved by the Leiden University Medical Centre ethical committee and an informed consent was signed by all participants prior to participation. Citrate plasma was available for 1644 offspring of nonagenarians and 734 controls. After quality control, plasma lipidome measurements were considered for 2201 participants, 1526 offspring of nonagenarian siblings (59 years ± 6.7), and 675 controls (59 years ± 7.5). Table 1 lists demographic characteristics of this population and previously reported parameters such as total triglycerides, lipoprotein particle size, body mass index (BMI), LDL-C, HDL-C, and prevalence of hypertension and diabetes (Mooijaart *et al*., 2006; Rozing *et al*., 2009; Westendorp *et al*., 2009; Rozing *et al*., 2010).

### Lipid profiling by LC-MS

Synthetic phospholipids, lysophosphatidylcholine LPC (17:0), LPC (19:0), PE (15:0/15:0), PE (17:0/17:0), PC (17:0/17:0), and PC (19:0/19:0) were obtained from Avanti Polar Lipids Inc. (Alabaster, AL, USA), triacylglycerols TG (51:0) and TG (45:0) were obtained from Sigma-Aldrich (Zwijndrecht, the Netherlands) and were added to plasma samples before lipid extraction as calibration and internal standards. Lipids from plasma aliquots (30 μL) were extracted according to the method of Bligh and Dyer with slight modifications (Bligh & Dyer, 1959).

Lipidomic analysis was performed using an optimized version of the method reported by Hu *et al*., (2008). Briefly, lipid extracts spiked with calibration and internal standards were separated by reverse-phase ultra-high pressure liquid chromatography coupled to mass spectrometry (UPLC-MS), using an Acquity UPLC system equipped with binary pump and autosampler at 15 °C (Waters, Milford, MA, USA). Lipids were separated in a T3 UPLC column 1.8 μm, 2.1 × 100 mm (Acquity Waters) at 55 °C using a dynamic gradient from 68% to 3% mobile phase A to mobile phase B in 17 min, followed by equilibration for a total run of 20 min. Mobile phase A consisted of water and acetonitrile (40:60% V/V), supplemented with 10 mm ammonium formate. Mobile phase B contained acetonitrile, isopropanol (10:90% V/V), and 10 mm ammonium formate. All solvents were UPLC grade (Biosolve, Valkenswaard, the Netherlands). The UPLC was coupled to a QToF mass spectrometer, 6530 Accurate Mass QToF-LC-MS (Agilent Technologies, Santa Clara, CA, USA) equipped with electrospray ionization with jet stream nebulizer, and Agilent Mass Hunter Acquisition software. Data were acquired using the following settings: gas temperature, 325 °C; nozzle voltage, 1000 V; gas flow, 10 L min^−1^; sheath gas flow, 12 L min^−1^; Vcap, 3500 V; mass range, 450–1600 m/z, in positive ion mode. The method was validated using plasma obtained with sodium citrate. Validation parameters were as follows: linearity LPC (19:0), *r*^2^ > 0.99; PC (17:0/17:0), *r*^2^ > 0.97; PE (17:0/17:0), *r*^2^ > 0.98; TG (45:0), *r*^2^ > 0.99; repeatability and reproducibility, RSD < 15%. Two freeze–thaw cycles did not alter validation parameters (RSD < 15%). After data acquisition, we used a targeted method and data mining to assess the relative levels of 150 lipids using MassHunter Quantitative Analysis Software (v.B.04.00; Agilent Technologies, 2008).

### Lipid nomenclature

Lipids names and abbreviations were assigned according to Lipid Maps nomenclature (http://www.lipidmaps.org). The analytical method determined total lipid composition as number of carbon atoms and double bonds without specifying the location of double bonds or the stereochemistry of the acyl chains, and differentiated the alkyl-acyl (ether)—from the diacyl—subclasses. The following accepted abbreviations were used: phosphocholines, PC; sphingomyelins, SM; triglycerides, TG; phosphoethanolamines, PE. The alkyl ether linkage is represented by the ‘O-’ prefix, for example, PC (O-34:1), TG (O-50:2), the numbers within parenthesis refer to the total number of carbons of the fatty acyl chains followed by the number of double bonds of all the chains.

### Lipid quantification

Relative ratios (RR) of each lipid species were obtained by dividing the area under the curve of each lipid by the area under the curve of its assigned internal standard. PC, PC-O, and SM species were assigned to the internal standard PC (17:0/17:0); LPC species—to the internal standard LPC (19:0); PE and LPE species–to the internal standard PE (17:0/17:0); cholesteryl ester (ChoE), diacylglycerol, and TG species—to the internal standard TG (51:0). Relative ratios (RR) were corrected for intra- and interbatch variation prior to statistical analyses (van der Kloet *et al*., 2009). Quality control: quality control samples (QC) prepared with pooled plasma from the same cohort were included every nine injections. In addition, randomly selected study samples (15%) were analyzed as independent duplicates. A double-quality-control approach was applied to include lipid species that were accurately measured throughout the study by considering only lipids species for which both duplicate and QC samples showed an RSD < 15% (*n* = 111) or RSD < 25% (*n* = 128).

### Statistical analyses

Most lipid species showed a right-skewed distribution thus, data were logarithmically transformed to approximate normality before statistical analyses.

For comparisons between offspring and controls, a linear regression model was fitted with lipid species as dependent variables and offspring/control status as a fixed factor. Data analyses were stratified by sex to assess sex-specific differences. Lipid species strongly correlated with BMI and age, and hence, these parameters were used as covariates. Also, a term for batch was included in the model to account for analytical batch effects.

To assess whether the association of lipid species linked to familial longevity was independent of total triglycerides, logistic regression analyses were performed in women with offspring/control status as the dependent variable and total triglycerides, age, BMI, and batch as independent variables. Family relationships were taken into account by including in the regression model a term to determine robust standard errors using the sandwich estimator, which considered data from one same family as a group. MUFA-to-PUFA ratios were calculated for the lipid species that associated with longevity by adding levels of all MUFA lipids (lipids with one double bond in any acyl chain), and the resulting value was divided by the sum of all PUFA lipids (species with two or more double bonds in their acyl chains), that is, ∑MUFA/∑PUFA. Lipid species, PC (O-36:2), and TG (57:2) were not considered in this calculation because the unknown location of the double bond made their classification as polyunsaturated or monounsaturated uncertain. MUFA-to-PUFA ratios were logarithmically transformed for further analyses. To test the effect of age after 55 years, a linear regression model was fitted with age before or after menopause (>55 or ≤55 years) as a dependent variable separately in offspring and controls. This study represents the first attempt to associate lipidomic profiles and familial longevity. Therefore, our exploratory approach defined statistical significance at an arbitrary threshold of *P*-value ≤0.005 for all analyses. Statistical analyses were performed using stata 11.0 (StataCorp LP, TX, USA, 2009) and r statistical programming system (http://www.r-project.org/). Graphics were created with graphpad Prism (version 4.0; La Jolla, CA, USA).

## Supporting Information

**Table S1** Demographics of the study population stratified by sex.

**Table S2.** Lipid species that associated with familial longevity in women.

**Table S3** Lipid species that associated with familial longevity in men.

**Table S4** Lipid species that associated with familial longevity in women independent of total triglycerides.

**Table S5** Effect of age in lipid species that associated with familial longevity in men and women.

**Table S6** Linear parameters of the relationship between age and levels of lipids that associated with familial longevity in women.
