# Supplementary material for: Lipidomics of familial longevity
Source: Aging Cell. 2013 Apr 2;12(3):426–34. doi: 10.1111/acel.12064 (PMC3709127; doi:10.1111/acel.12064)
Supplement: Supplementary file 1 [file acel0012-0426-SD1.doc]

Table S1. Demographics of the Study Population Stratified by Sex

|  | All | | Females | |
| --- | --- | --- | --- | --- |
|  | Controls | Offspring | Controls | Offspring |
| N | 675 | 1525 | 390 | 812 |
| Age (years) | 58.8 ± 7.4 | 59.3 ± 6.6 | 56.9 ± 6.8 | 59.3 ± 6.5 |
| BMI (kg/m2) | 25.6 ± 3.6 | 25.3 ± 3.6 | 25.4 ± 3.8 | 25.0 ± 4.0 |
| TG (mmol/L) | 1.94 ± 1.37 | 1.76 ± 1.05 | 1.74 ± 1.06 | 1.55 ± 0.860 |
| HDL-C (mmol/L) | 1.41 ± 0.465 | 1.44 ± 0.448 | 1.55 ± 0.491 | 1.59 ± 0.459 |
| LDL-C (mmol/L) | 3.36 ± 0.938 | 3.34 ± 0.987 | 3.39 ± 0.939 | 3.38 ± 1.03 |
| HDL particle size (nm) | 9.01 ± 0.509 | 9.05 ± 0.507 | 9.18 ± 0.506 | 9.25 ± 0.469 |
| LDL particle size (nm) | 21.1 ± 0.388 | 21.2 ± 0.826 | 21.4 ± 0.797 | 21.5 ± 0.750 |
| TG/HDLC ratio | 1.66 ± 1.54 | 1.45 ± 1.27 | 1.37 ± 1.27 | 1.11 ± 0.909 |
| Diabetes type 2 (%) | 8.2 ± 1.1 | 4.3 ± 0.56 | 6.0 ± 1.3 | 3.6 ± 0.71 |
| Hypertension (%) | 28.7 ± 1.9 | 22.3 ± 1.2 | 28.9 ± 2.5 | 22.1 ± 1.6 |
| MUFA-to-PUFA ratio | 0.881 ± 0.37 | 0.927 ± 0.380 | 0.917 ± 0.350 | 1.00 ± 0.341 |

Data is reported as mean values ± SD. ^a^Data is reported as percentage of cases ± SD. ^b^Values are adjusted means from the regression model ± SD.

Table S2. Lipid species that associated with familial longevity in females

| Lipid | Controls^a^ | Offspring^a^ | Beta ± SE^b^ | P-value^c^ |
| --- | --- | --- | --- | --- |
| PC (O-34:3) | 1.474 | 1.572 | 0.064 ± 0.019 | 6.1x10^-4^ |
| PC (O-34:1) | 0.618 | 0.651 | 0.053 ± 0.015 | 2.7x10^-4^ |
| PC (O-36:3) | 0.275 | 0.296 | 0.073 ± 0.020 | 2.0x10^-4^ |
| PC (O-36:2) | 0.183 | 0.199 | 0.087 ± 0.020 | 1.4x10^-5^ |
| PE (38:6) | 1.197 | 1.085 | -0.098 ± 0.033 | 3.2x10^-3^ |
| SM (d18:1/14:0) | 0.351 | 0.373 | 0.061 ± 0.016 | 1.4x10^-4^ |
| SM (d18:1/15:0) | 0.209 | 0.223 | 0.066 ± 0.016 | 2.8x10^-5^ |
| SM (d18:1/16:0) | 6.943 | 7.184 | 0.034 ± 0.011 | 2.8x10^-3^ |
| SM (d18:1/17:0) | 0.072 | 0.076 | 0.054 ± 0.019 | 3.6x10^-3^ |
| SM (d18:1/18:2) | 0.035 | 0.037 | 0.054 ± 0.019 | 5.0x10^-3^ |
| SM (d18:1/21:0) | 0.316 | 0.337 | 0.066 ± 0.017 | 8.9x10^-5^ |
| SM (d18:1/23:1) | 0.573 | 0.601 | 0.049 ± 0.014 | 5.6x10^-4^ |
| SM (d18:1/23:0) | 0.852 | 0.886 | 0.040 ± 0.014 | 4.9x10^-3^ |
| TG (52:1) | 1.626 | 1.457 | -0.110 ± 0.035 | 2.0x10^-3^ |
| TG (54:7) | 0.165 | 0.147 | -0.121 ± 0.038 | 1.4x10^-3^ |
| TG (54:6) | 0.510 | 0.447 | -0.133 ± 0.040 | 9.7x10^-4^ |
| TG (56:7) | 0.470 | 0.416 | -0.122 ± 0.041 | 3.3x10^-3^ |
| TG (56:6) | 0.521 | 0.472 | -0.098 ± 0.031 | 1.7x10^-3^ |
| TG (57:2) | 0.545 | 0.490 | -0.106 ± 0.031 | 6.9x10^-4^ |

^a^ Back-transformed adjusted means of relative lipid levels; ^b^ Beta, effect size of lipid levels and robust standard error. Positive values indicate higher levels in offspring of nonagenarians and negative values indicate lower levels in offspring compared to controls; ^c^ Nominal P-value.

Table S3. Lipid species that associated with familial longevity in males

| Lipid | Controls^a^ | Offspring^a^ | Beta ± SE^b^ | P-value^c^ |
| --- | --- | --- | --- | --- |
| PC (O-34:3) | 1.221 | 1.262 | 0.033 ± 0.021 | 0.111 |
| PC (O-34:1) | 0.505 | 0.515 | 0.019 ± 0.016 | 0.239 |
| PC (O-36:3) | 0.252 | 0.257 | 0.021 ± 0.023 | 0.378 |
| PC (O-36:2) | 0.165 | 0.173 | 0.045 ± 0.024 | 0.067 |
| PE (38:6) | 0.493 | 0.471 | -0.044 ± 0.042 | 0.305 |
| SM (d18:1/14:0) | 0.399 | 0.402 | 0.007 ± 0.020 | 0.715 |
| SM (d18:1/15:0) | 0.260 | 0.264 | 0.016 ± 0.020 | 0.401 |
| SM (d18:1/16:0) | 7.677 | 7.708 | 0.004 ± 0.013 | 0.765 |
| SM (d18:1/17:0) | 0.104 | 0.105 | 0.003 ± 0.019 | 0.895 |
| SM (d18:1/18:2) | 0.038 | 0.039 | 0.019 ± 0.021 | 0.380 |
| SM (d18:1/21:0) | 0.397 | 0.402 | 0.011 ± 0.021 | 0.614 |
| SM (d18:1/23:1) | 0.706 | 0.713 | 0.010 ± 0.018 | 0.591 |
| SM (d18:1/23:0) | 1.020 | 1.020 | 2.0x10^4^ ± 0.017 | 0.991 |
| TG (52:1) | 1.931 | 1.839 | -0.049 ± 0.042 | 0.239 |
| TG (54:7) | 0.252 | 0.250 | -0.007 ± 0.047 | 0.881 |
| TG (54:6) | 1.245 | 1.198 | -0.039 ± 0.049 | 0.436 |
| TG (56:7) | 0.643 | 0.616 | -0.043 ± 0.047 | 0.354 |
| TG (56:6) | 0.604 | 0.574 | -0.051 ± 0.031 | 0.197 |
| TG (57:2) | 0.659 | 0.628 | -0.048 ± 0.038 | 0.216 |

^a^ Back-transformed adjusted means of relative lipid levels; ^b^ Beta, effect size of lipid levels and robust standard error; positive values indicate higher levels in offspring of nonagenarians and negative values indicate lower levels in offspring compared to controls; ^c^ Nominal P-value.

Table S4. Lipid species that associated with familial longevity in females independent of total triglycerides

| Lipid | Beta ± SE^a^ | P-value^b^ |
| --- | --- | --- |
| PC (O-34:3) | 0.719 ± 0.274 | 0.009 |
| PC (O-34:1) | 0.958 ± 0.352 | 0.006 |
| PC (O-36:3) | 0.754 ± 0.244 | 0.002 |
| PC (O-36:2) | 0.928 ± 0.241 | 1.1x10^-4^ |
| PE (38:6) | -0.308 ± 0.156 | 0.049 |
| SM (d18:1/14:0) | 1.172 ± 0.307 | 1.3x10^-4^ |
| SM (d18:1/15:0) | 1.176 ± 0.303 | 1.0x10^-4^ |
| SM (d18:1/16:0) | 1.031 ± 0.428 | 0.016 |
| SM (d18:1/17:0) | 0.618 ± 0.371 | 0.096 |
| SM (d18:1/18:2) | 0.646 ± 0.266 | 0.015 |
| SM (d18:1/21:0) | 1.096 ± 0.280 | 9.0x10^-4^ |
| SM (d18:1/ 23:1) | 0.924 ± 0.322 | 0.004 |
| SM (d18:1/23:0) | 0.886 ± 0.330 | 0.007 |
| TG (52:1) | -0.163 ± 0.185 | 0.379 |
| TG (54:7) | -0.293 ± 0.142 | 0.039 |
| TG (54:6) | -0.277 ± 0.142 | 0.060 |
| TG (56:7) | -0.281 ± 0.175 | 0.107 |
| TG (56:6) | -0.305 ± 0.187 | 0.104 |
| TG (57:2) | -0.280 ± 0.166 | 0.092 |

^a^ Beta, effect size of lipid levels and robust standard error from logistic regression analysis; positive values indicate higher levels in the offspring of nonagenarians and negative values indicate lower levels in offspring compared to controls; ^b^ Nominal P-value.

Table S5. Effect of age in lipid species that associated with familial longevity in males versus females

|  | Females  N = 1202 | | Males  N = 999 | |
| --- | --- | --- | --- | --- |
| Lipid | Beta ± SE^b^ | P-value^c^ | Beta ± SE^b^ | P-value^c^ |
| PC (O-34:3) | -0.076 ± 0.013 | 9.07x10^-09^ | -0.060 ± 0.014 | 2.7x10^-5^ |
| PC (O-34:1) | 0.034 ± 0.010 | 9.10x10^-04^ | -0.009 ± 0.011 | 0.427 |
| PC (O-36:3) | -0.049 ± 0.015 | 7.22x10^-04^ | -0.037 ± 0.015 | 0.015 |
| PC (O-36:2) | -0.044 ± 0.016 | 5.00x10^-03^ | -0.017 ± 0.016 | 0.281 |
| PE (38:6) | 0.042 ± 0.023 | 7.17x10^-02^ | 0.058 ± 0.029 | 0.049 |
| SM (d18:1/14:0) | 0.052 ± 0.012 | 1.62x10^-05^ | 0.017 + 0.013 | 0.179 |
| SM (d18:1/15:0) | 0.064 ± 0.012 | 9.47x10^-08^ | 0.029 ± 0.013 | 0.036 |
| SM (d18:1/16:0) | 0.020 ± 0.008 | 9.18x10^-03^ | -0.001 ± 0.001 | 0.929 |
| SM (d18:1/17:0) | 0.082 ± 0.014 | 2.46x10^-09^ | 0.020 ± 0.014 | 0.135 |
| SM (d18:1/18:2) | 0.003 ± 0.014 | 8.43x10^-01^ | -0.002 ± 0.014 | 0.883 |
| SM (d18:1/21:0) | 0.042 ± 0.013 | 1.04x10^-03^ | -0.009 ± 0.015 | 0.566 |
| SM (d18:1/23:1) | 0.051 ± 0.011 | 2.85x10^-06^ | 0.006 ± 0.012 | 0.616 |
| SM (d18:1/23:0) | 0.022 ± 0.010 | 3.11x10^-02^ | -0.024 ± 0.012 | 0.024 |
| TG (52:1) | 0.098 ± 0.024 | 5.42x10^-05^ | 0.052 ± 0.028 | 0.065 |
| TG (54:7) | 0.109 ± 0.027 | 6.55x10^-05^ | 0.023 ± 0.034 | 0.493 |
| TG (54:6) | 0.097 ± 0.028 | 6.82x10^-04^ | -0.090 ± 0.037 | 0.813 |
| TG (56:7) | 0.082 ± 0.027 | 2.52x10^-03^ | 0.025 ± 0.032 | 0.431 |
| TG (56:6) | 0.088 ± 0.023 | 1.30x10^-04^ | -0.009 ± 0.028 | 0.735 |
| TG (57:2) | 0.071 ± 0.023 | 2.45x10^-03^ | 0.052 ± 0.027 | 0.053 |

^a^ Beta, effect size of lipid levels and robust standard error from linear regression analysis; positive values indicate higher levels in the offspring of nonagenarians and negative values indicate lower levels in offspring compared to controls; ^b^ Nominal P-value.

Table S6. Linear parameters of the relationship between age and levels of lipid species that associated with familial longevity in females

|  | Offspring | | | | | | | | |
| --- | --- | --- | --- | --- | --- | --- | --- | --- | --- |
|  | All ages (812) | | | ≤55 years (200) | | | >55 years (612) | | |
|  | B | Slope | R | B | Slope | R | B | Slope | R |
| PC (O-34:3) | 0.227 | -0.0097 | -0.224* | 0.135 | -0.0078 | -0.089 | 0.169 | -0.0088 | -0.154* |
| PC (O-34:1) | -0.500 | -0.0058 | -0.174* | -0.444 | -0.0067 | -0.108 | -0.568 | -0.0047 | -0.104 |
| PC (O-36:3) | -1.179 | -0.0078 | -0.166* | -0.971 | -0.0116 | -0.117 | -1.323 | -0.0055 | -0.090 |
| PC (O-36:2) | -1.695 | -0.0077 | -0.163* | -1.214 | -0.0168 | -0.175 | -1.932 | -0.0040 | -0.064 |
| PE (38:6) | 0.199 | 0.0029 | 0.038 | -0.455 | 0.0160 | 0.096 | 0.148 | 0.0036 | 0.037 |
| SM (d18:1/14:0) | -0.744 | 0.0041 | 0.114* | -1.141 | 0.0117 | 0.157 | -0.616 | 0.0021 | 0.043 |
| SM (d18:1/15:0) | -1.446 | 0.0057 | 0.157* | -1.669 | 0.0097 | 0.134 | -1.261 | 0.0028 | 0.058 |
| SM (d18:1/16:0) | 2.027 | 0.0004 | 0.016 | 1.821 | 0.0042 | 0.086 | 2.165 | -0.0017 | -0.050 |
| SM (d18:1/17:0) | -2.367 | 0.0067 | 0.151* | -2.075 | 0.0003 | 0.002 | -2.078 | 0.0022 | 0.044 |
| SM (d18:1/18:2) | -3.128 | 0.0016 | 0.039 | -3.104 | 0.0009 | 0.011 | -3.012 | -0.0002 | -0.004 |
| SM (d18:1/21:0) | -0.889 | 0.0029 | 0.073 | -1.131 | 0.0073 | 0.089 | -0.714 | 0.0001 | 0.002 |
| SM (d18:1/23:1) | -0.346 | 0.0031 | 0.092 | -0.772 | 0.0110 | 0.162 | -0.090 | -0.0009 | -0.021^a^ |
| SM (d18:1/23:0) | 0.066 | 0.0005 | 0.016 | -0.301 | 0.0073 | 0.105 | 0.330 | -0.0036 | -0.083 |
| TG (52:1) | 1.280 | 0.0104 | 0.122* | 0.124 | 0.0329 | 0.191* | 1.525 | 0.0065 | 0.057 |
| TG (54:7) | -1.236 | 0.0114 | 0.135* | -2.256 | 0.0312 | 0.176 | -1.016 | 0.0079 | 0.071 |
| TG (54:6) | 0.003 | 0.0122 | 0.134* | -1.485 | 0.0410 | 0.219* | 0.374 | 0.0063 | 0.052^a^ |
| TG (56:7) | -0.104 | 0.0101 | 0.120* | -0.894 | 0.0251 | 0.140 | 0.209 | 0.0051 | 0.047 |
| TG (56:6) | -0.081 | 0.0118 | 0.165* | -1.083 | 0.0313 | 0.205* | 0.101 | 0.0089 | 0.094 |
| TG (57:2) | -0.057 | 0.0079 | 0.096* | -0.793 | 0.0223 | 0.136 | 0.035 | 0.0064 | 0.058 |

Linear parameters: B, line intercept at age = 0; R, Pearson correlation coefficient; * Pearson correlation coefficient, P<0.005 and slopes are different from zero, P<0.005. ^a^ Differences in slopes between offspring and controls were statistical significant, P<0.005.

Continued, Table S6

|  | Controls | | | | | | | | |
| --- | --- | --- | --- | --- | --- | --- | --- | --- | --- |
|  | All ages (390) | | | ≤55 years (147) | | | >55 years (243) | | |
|  | B | Slope | R | B | Slope | R | B | Slope | R |
| PC (O-34:3) | 0.074 | -0.0081 | -0.191* | -0.385 | 0.0017 | 0.028 | -0.348 | -0.0016 | -0.023 |
| PC (O-34:1) | -0.662 | -0.0039 | -0.124 | -0.728 | -0.0021 | -0.044 | -1.112 | 0.0032 | 0.065 |
| PC (O-36:3) | -1.387 | -0.0052 | -0.122 | -1.976 | 0.0074 | 0.108 | -1.961 | 0.0037 | 0.057 |
| PC (O-36:2) | -2.022 | -0.0035 | -0.081 | -2.584 | 0.0086 | 0.138 | -2.555 | 0.0048 | 0.070 |
| PE (38:6) | 0.036 | 0.0072 | 0.099 | 0.897 | -0.0106 | -0.093 | -0.003 | 0.0082 | 0.070 |
| SM (d18:1/14:0) | -0.979 | 0.0074 | 0.214* | -1.059 | 0.0091 | 0.171 | -1.110 | 0.0094 | 0.171 |
| SM (d18:1/15:0) | -1.615 | 0.0077 | 0.220* | -1.590 | 0.0075 | 0.147 | -1.958 | 0.0132 | 0.225* |
| SM (d18:1/16:0) | 1.873 | 0.0025 | 0.104 | 1.755 | 0.0051 | 0.142 | 1.706 | 0.0051 | 0.127 |
| SM (d18:1/17:0) | -2.620 | 0.0103 | 0.261* | -2.663 | 0.0113 | 0.195 | -2.782 | 0.0128 | 0.200* |
| SM (d18:1/18:2) | -3.114 | 0.0007 | 0.016 | -2.755 | -0.0065 | -0.099 | -3.344 | 0.0045 | 0.067 |
| SM (d18:1/21:0) | -1.174 | 0.0070 | 0.191* | -1.382 | 0.0115 | 0.219 | -1.440 | 0.0111 | 0.182* |
| SM (d18:1/23:1) | -0.634 | 0.0074 | 0.236* | -0.654 | 0.0080 | 0.171 | -0.824 | 0.0104 | 0.204*^a^ |
| SM (d18:1/23:0) | -0.202 | 0.0046 | 0.156* | -0.430 | 0.0094 | 0.221 | -0.233 | 0.0050 | 0.101 |
| TG (52:1) | 1.371 | 0.0109 | 0.139* | 1.587 | 0.0063 | 0.052 | 1.537 | 0.0083 | 0.065 |
| TG (54:7) | -1.241 | 0.0138 | 0.161* | -0.721 | 0.0032 | 0.025 | -1.393 | 0.0164 | 0.116 |
| TG (54:6) | 0.230 | 0.0110 | 0.120 | 0.662 | 0.0020 | 0.015 | 0.302 | 0.0100 | 0.066^a^ |
| TG (56:7) | -0.026 | 0.0111 | 0.120 | 0.638 | -0.0026 | -0.022 | -0.048 | 0.0117 | 0.072 |
| TG (56:6) | 0.202 | 0.0088 | 0.124 | 0.973 | -0.0072 | -0.071 | 0.274 | 0.0080 | 0.067 |
| TG (57:2) | -0.026 | 0.0092 | 0.141* | 0.610 | -0.0038 | -0.039 | -0.170 | 0.0117 | 0.109 |

Linear parameters: B, line intercept at age = 0; R, Pearson correlation coefficient; * Pearson correlation coefficient, P<0.005 and slopes are different from zero, P<0.005. ^a^ Differences in slopes between offspring and controls were statistical significant, P<0.005.
